# Evolutionary genomics of *LysM *genes in land plants

**DOI:** 10.1186/1471-2148-9-183

**Published:** 2009-08-03

**Authors:** Xue-Cheng Zhang, Steven B Cannon, Gary Stacey

**Affiliations:** 1 Division of Plant Sciences and National Center for Soybean Biotechnology, University of Missouri, Columbia, MO 65211 USA; 2 USDA-ARS Corn Insect and Crop Genetics Research Unit, and Department of Agronomy, Iowa State University, Ames, IA 50011 USA; 3 Center for Sustainable Energy, Division of Biochemistry, Department of Molecular Microbiology and Immunology, University of Missouri, Columbia, MO 65211 USA

## Abstract

**Background:**

The ubiquitous LysM motif recognizes peptidoglycan, chitooligosaccharides (chitin) and, presumably, other structurally-related oligosaccharides. LysM-containing proteins were first shown to be involved in bacterial cell wall degradation and, more recently, were implicated in perceiving chitin (one of the established pathogen-associated molecular patterns) and lipo-chitin (nodulation factors) in flowering plants. However, the majority of *LysM *genes in plants remain functionally uncharacterized and the evolutionary history of complex *LysM *genes remains elusive.

**Results:**

We show that LysM-containing proteins display a wide range of complex domain architectures. However, only a simple core architecture is conserved across kingdoms. Each individual kingdom appears to have evolved a distinct array of domain architectures. We show that early plant lineages acquired four characteristic architectures and progressively lost several primitive architectures. We report plant *LysM *phylogenies and associated gene, protein and genomic features, and infer the relative timing of duplications of *LYK *genes.

**Conclusion:**

We report a domain architecture catalogue of LysM proteins across all kingdoms. The unique pattern of LysM protein domain architectures indicates the presence of distinctive evolutionary paths in individual kingdoms. We describe a comparative and evolutionary genomics study of *LysM *genes in plant kingdom. One of the two groups of tandemly arrayed plant *LYK *genes likely resulted from an ancient genome duplication followed by local genomic rearrangement, while the origin of the other groups of tandemly arrayed *LYK *genes remains obscure. Given the fact that no animal *LysM *motif-containing genes have been functionally characterized, this study provides clues to functional characterization of plant *LysM *genes and is also informative with regard to evolutionary and functional studies of animal *LysM *genes.

## Background

The Lysin motif (LysM), usually 42–48 amino acids in length, is a ubiquitous modular cassette found in virtually every living organism except for Archaea [1,2]. X-ray crystallography and homology-modeling of LysM motifs revealed a symmetrical βααβ topology with the two α helices residing on one side of a two-stranded antiparallel β-sheet [1,3,4]. A detailed domain folding study indicated that the LysM domain folds in a stepwise and robust manner [5]. In bacteria, LysM proteins are generally involved in bacterial cell wall degradation by anchoring enzymatic domains to the cell wall through binding to peptidoglycan (PGN), a linear form of alternatively β1→4 linked N-acetyl-muramic acid and N-acetyl-glucosamine (GlcNAc) [6,7]. A LysM-containing receptor-like protein (LYP) (lacking a kinase domain), CEBiP, was shown in rice to biochemically bind chitin, a β1→4 linked homopolymer of N-acetylglucosamine [8]. In Arabidopsis, LysM-containing receptor-like kinases (LYKs) were genetically defined as receptors for chitin [9,10]. Chitin is a major component of the fungal cell wall and an established pathogen-associated molecular pattern (PAMP). Hence, these LYKs were implicated in the plant defense response to fungal pathogens. In contrast, other LYKs (e.g., NFR1, NFR5) were genetically defined as receptors for the rhizobial nodulation factor [11-13], an acylated, substituted chitin oligomer. In this case, these receptors are coupled to a complex plant developmental pathway leading to the formation of a novel organ, the nodule, in which nitrogen fixing, symbiotic bacteria reside. Although no direct evidence exists in vertebrates, the LysM domain is presumably capable of binding peptidoglycan, chitin and structurally-related oligosaccharide molecules in these organisms.

In a previous study, we categorized LysM motifs across kingdoms into a minimum of 11 distinctive types [2]. The phylogenetic gene family topology based on LysM motif sequences revealed several multiple-kingdom clades. Interestingly, several bacterial-rooted LysM clades contained sequences from fungi, insects, plants and animals. This suggested that some LysM motif patterns may have origins predating the divergence of fungal, plant and animal kingdoms, while other LysM motifs may have originated in a convergent manner [2]. Besides the great diversity among LysM motifs, the numbers of LysM motifs within individual LysM proteins range from one to twelve. Moreover, LysM motifs are often associated with a diversity of other protein domains. The diversity of the LysM motifs and associated domains makes it possible to create a catalog of distinguishable LysM protein domain architectures. In the Pfam database, LysM motifs are associated with over 50 domains and LysM proteins display 241 domain architectures . Although the LysM domain is associated with a variety of protein domains across kingdoms, it is intriguing that some associations are distinct to particular kingdoms. For example, LYK proteins are only present in plants [1,2]. Nevertheless, the evolutionary dynamics of LysM protein architectures in individual kingdoms, especially the plant kingdom, have not been clearly defined.

The last six years have seen a great leap forward in our understanding of the biological functions of plant LysM proteins. At the same time, a large number of uncharacterized plant LysM-protein sequences were deposited in public databases. Little is known regarding the origins, evolution, common gene and protein features, and comparative genomics of these plant *LysM *genes. A multiple-dimension "atlas" (within and across species, considering phylogenetic context) of plant *LysM *genes is needed to better understand this important gene family in the entire plant kingdom.

The ubiquity of *LysM *genes across kingdoms, especially within the plant kingdom, makes it an appropriate candidate gene family to study the evolution and impact of polyploidy on plant genomes. The genomes of soybean (*Glycine max*) and poplar (*Populus trichocarpa*) have a greater number of *LYK *genes than Arabidopsis, rice and *Medicago truncatula*, probably due to the influence of an additional genome duplication in each of the *Glycine *and *Populus *genera. Tandemly arrayed *LYK *gene pairs were identified in legume and poplar plants, but not in rice, suggesting that these tandem gene duplications or local genome rearrangements occurred only in certain plant lineages [2].

In this study, we report a domain architecture catalogue of LysM proteins across kingdoms and reconstruct evolutionary scenarios of *LysM *genes in the plant kingdom. While a simple core domain architecture of LysM proteins is conserved across kingdoms, each individual kingdom has a unique array of domain architectures. Compared to green algae such as *Chlamydomonas*, the moss *Physcomitrella *possesses a more diverse set of LysM proteins, including LYK, LYP, and LysM-containing F-box proteins. More importantly, these LysM architectures persist throughout all major plant lineages. In contrast, gymnosperms appear to have lost diversity among the LysM proteins. We calculated majority-ruled parsimony phylogenies of plant LysM genes and present associated gene, protein and genomic features along with the phylogenies. We also investigated the relative timing and patterns of large-scale duplications of *LYK *genes. Our analysis shows that a block of tandemly arrayed *LYK *genes likely resulted from an ancient genome duplication followed by local genome rearrangements. This study will provide clues to functional characterization of plant *LysM *genes and be informative to functional studies of animal *LysM *genes.

## Results

### LysM domain architectures across kingdoms

Although the LysM domain family is relatively small compared to the top 20 PFAM domain families in terms of the number of sequences, it displays a much wider range of domain architectures than 10 of the top 20 domain families . The association of LysM with a kinase domain appears to be present only in the plant kingdom. However, it remains unclear whether other individual kingdoms possess unique domain architectures. To this end, we surveyed the LysM domain arrangements of over 5600 sequences curated in the Pfam database and drew all architectures represented by more than 5 sequences (3ure 1). The simplest architecture, containing only one LysM motif, is present in all kingdoms. Consistent with the inter-kingdom conservation of this type of LysM protein, the LysM motifs of this family are also conserved, as revealed by sequence alignment, and they form a complete clade in a parsimony phylogeny that is distinguishable from other LysM-motif clades [2]. Proteins containing 2 LysM motifs and a peptidase domain are present in prokaryotes, green algae, and *Physcomitrella*, a non-vascular plant. Indeed these two architectures are the most common, comprising about one third of the LysM sequences curated in the Pfam database. Similar to the unique occurrence of LYK in the plant kingdom, a few architectures are exclusively present in individual kingdoms; e.g., amidase+xLysM and SLT+xLysM in bacteria; LysM+glycol_hydro_18 and LysM+chitin binding+glycol_hydro_18 in fungi; and LysM+GRAM, LysM+TLD, and LysM+GRAM+TLD in animals. This suggests that LysM proteins in different kingdoms evolved specialized architectures and likely distinct functions. Moreover, compared to other kingdoms, the prokaryotic kingdom displays more diversified LysM domain architectures, which is reflected by the variation in the numbers and the sequences of LysM motifs and the domains associated with the LysM domain. Plant LysM proteins, except a LysM protein in *Selaginella *(jgi|Selmo1|416289|fgenesh2_pg.C_scaffold_30000050) having 4 LysM motifs, contains no more than 3 copies of the LysM motifs. Like prokaryotic LysM proteins, multiple LysM domains are always distinct. In contrast, LysMs in fungi and animals are usually similar at the level of primary sequences. An extraordinary example is a nematode gene (NP_504862), which contains 12 nearly identical tandem LysM motifs.

**Figure 1:**
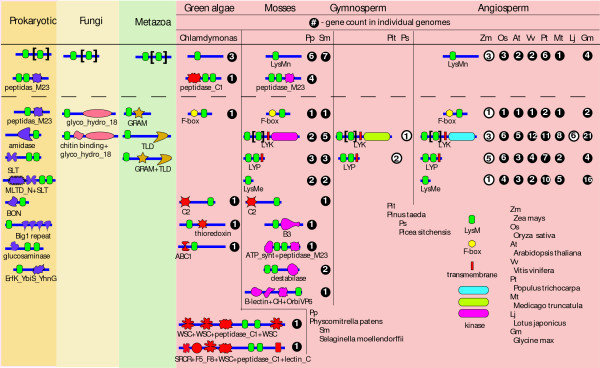
**The domain catalogue of LysM proteins across kingdoms**. Only domain architectures with more than 5 occurrences in the Pfam database were diagramed roughly to scale. Domains were represented as different symbols and domain names were labeled beneath the diagrams except for the LysM domain, which is not labeled. The domain architectures above the dash line are conserved across different kingdoms and those below the dash line are distinct to each kingdom. The black brackets denote that the numbers of LysM are variable, ranging from 0 to 11 in prokaryotes, fungi and animal kingdoms and ranging from 0 to 2 in plant LYK proteins. The apparent lack of architectural diversity in *Pinus *is most likely due to the incomplete genome sequence of this species. The predicted subcellular localizations of LysM proteins in plants are also illustrated by drawing the transmembrane domain and drawing the diagram from N- to C-terminus. The incidence of individual architectures in plants is labeled on the right of the diagram. The numbers in filled circles denote the overall architectures in the entire genome and those in open circles are incomplete due to the lack of sufficient genome sequences.

Besides LYK, we identified other architectures that are unique to and common in the plant kingdom (Figure 1). The F-box+LysM combination is found not only in mosses and angiosperm plants but also in green algae. LYP and extracellular LysM (LysMe) are the other two common architectures that are unique in plants. Interestingly, angiosperm plants have apparently lost a few architectures present in green algae and mosses, such as peptidase+LysM; C2+LysM; LysM+B3; and LysM+thioredoxin (Figure 1). This suggests that overall gross diversity of LysM protein architectures has decreased in angiosperm plants.

The copy numbers of intracellular non-secretory LysM genes (LysMn) are similar in the ten plant genomes we examined. The same also holds true for F-box+LysM genes, and *LYP *genes (Figure 1). In particular, the F-box+LysM gene appears to be single-copy in these plant genomes, except for poplar and soybean, each with two copies (Figure 1). In contrast, there is clearly an expansion of the *LYK *gene family in virtually all genomes examined, and of the *LysMe *genes in the poplar and soybean genomes. The *LYK *gene family has expanded in the angiosperms; for example, there are 2 *LYKs *in *Physcomitrella *(bryophytes), 5 *LYKs *in the *Selaginella *(lycophytes), and 21 *LYKs *in *Glycine max *(Figure 1).

### Phylogenies and comparative genomics of *LysM *genes

**Figure 2:**
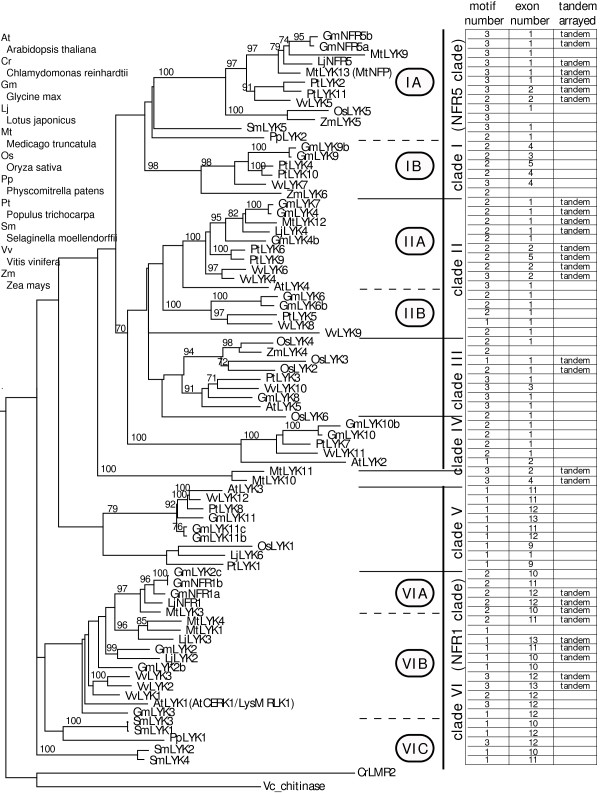
**Phylogenetic topology of plant LYK proteins**. A majority-ruled parsimony tree with maximum-likelihood branch lengths was calculated using full-length LYK sequences. Bootstrap values of 1000 independent trees larger than 70 were labeled on each branch. The tree was rooted using two algae sequences, Vc_chitinase (AAC13727) and CrLMR2 (EDP05962). Major clades are separated by solid horizontal lines and subclades are separated by dashed lines. On the right, the numbers of the LysM motifs are aligned in the first column, followed by the numbers of exons in the middle column, and tandemly arrayed genes are labeled in the third column.

We examined the phylogenetic relationships of the LYK, LYP, LysMe and LysMn protein families by calculating majority-rule parsimony topologies based on the full-length protein sequences. We grouped a total of 76 LYKs from 10 species into 6 multi-plant-family clades and a small group containing only MtLYK10 and 11 (Figure 2). Clades I and II share a similar topology and species composition. Interestingly, LYKs in the "primitive" plant genera, *Physcomitrella *and *Selaginella*, are found in clades I and VI. PpLYK1 and PpLYK2, located at the basal branches of clades I and VI. Clades I and VI are "complete" in the sense that they contain sequences from all sampled plant lineages. Most of the legume LYKs in subclades VIA and IA were genetically defined as the putative receptors of nodulation factors. Yet the non-legume LYKs orthologous to these putative nodulation factor receptors are functionally undefined. This arrangement suggests a structural, and presumed functional, conservation in plants, with more recent specialization in legumes for nodulation factor recognition. One functional possibility for the non-legume LYK proteins in clade I is as receptors for a postulated signal required for mycorrhizal infection. For example, it is interesting to note that there are no Arabidopsis *LYK *genes in clade I, consistent with the fact that this plant does not establish a symbiotic relationship with mycorrhiza. In addition to the nodulation factor receptors, the Arabidopsis chitin receptor is also found in clade VI, consistent with the notion that chitin recognition is a more ancient trait from which nodulation factor recognition evolved [2].

In order to better illustrate the relationships among the various LYK proteins, Figure 2 also shows the gene and genomic features of each clade in conjunction with the phylogenetic tree (Figure 2). Most LYKs in subclade IA have a conserved domain architecture, with 3 LysM motifs and a non-functional kinase domain lacking the P-loop and the activation motif [2]. Similarly, clade V contains only one LysM motif. Most *LYKs *in clade I and II have a simple gene structure with only one exon, while *LYKs *in clade V and VI possess a complex gene structure with more than 10 exons. However, most of these introns are embedded in the region encoding for the kinase domain, while the LysM domains are encoded by an uninterrupted exon (data not shown). Interestingly, 28 out of 79 *LYKs *in this study are tandemly arrayed in several genomes [2]. Namely, 35% of *LYKs *are tandemly arrayed, which is higher than the average level of tandemly arrayed genes in Arabidopsis, (~16%), rice (~14%) and poplar (11%) [14-16]. Subclade IA LysMs are oriented head-to-head with LysMs in subclade IIA, while LYKs in subclade VIA are oriented in a head-to-tail manner with LYKs in subclade VIB [2]. However, tandemly arrayed LYKs were not identified in monocots, suggesting that tandem duplication of these genes may have occurred after the split of monocot and dicot plants.

**Figure 3:**
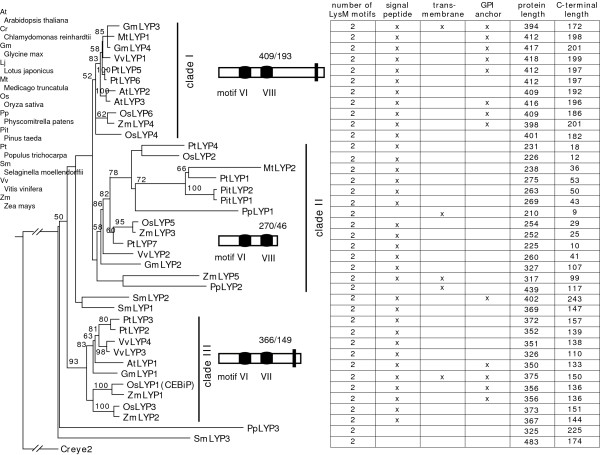
**Phylogenetic topology of plant LYP proteins**. A majority-ruled parsimony tree with maximum-likelihood branch lengths was calculated using full-length LYP sequences. Bootstrap values of 1000 independent trees larger than 50 are labeled on each branch. The tree was rooted using the algae sequence Creye2 (AAF43040). Detailed diagrams of each LYP clade are drawn. The classification types of LysM motifs described in Zhang et al. (2007) are shown along with the diagrams. The vertical bar denotes the presence of either a transmembrane domain or a predicted GPI anchor site. Note the correlation between the length of the whole protein and the length of the C-terminal tails.

We grouped a total of 39 LYPs from 9 species into 3 clades (Figure 3). The clades I and III contain LYPs exclusively from angiosperm plants, while the clade II contains LYPs from both diverse eudicots and monocots as well as LYPs from *P. Taeda *and *P. patens*. Compared to clades I and III, sequences in clade II have a higher rate of coding changes, as evidenced by the longer maximum-likelihood branch lengths on the topology (Figure 3). All LYPs have exactly 2 LysM motifs without exceptions. At the primary sequence level and dictated by their phylogenetic topology (data not shown), the first LysM motif, designated as motif A, of all LYPs is highly similar and was previously denoted as the plant LysM type VI [2]. In contrast, the second motif, designated as motif B, diverges. The motif B in clade I and II, denoted as plant LysM motif VIII, is different from that in clade III denoted as plant LysM motif VII [2]. The majority of LYPs contain a signal peptide. Although a few have a predicted transmembrane domain, about one third of them, especially LYPs in clade I, have a predicted GPI anchor that may function to anchor the protein to the plasma membrane (Figure 3). Therefore, about half of the LYP are predicted to be directly or indirectly localized to the plasma membrane. In terms of amino acid (aa) lengths, clade I LYPs generally are the longest (mean ± SE, 409 ± 2.4), followed by clade III LYPs (366 ± 11.7), while clade II LYPs are the shortest (270 ± 15.7). Despite the difference in their aa length, the position of the LysM motifs are relatively fixed with motif A at about position 110–150 and motif B at about position 170–220 (data not shown). Therefore, the lengths of the C-terminal tail are directly proportional to full protein length. Generally, the clade I tails (193 ± 2.8) contain a predicted GPI anchor, only a few of the clade III tails (149 ± 8.2) have a predicted GPI anchor, and the clade II tails (46 ± 9.7) lack a predicted GPI anchor (Figure 3.).

**Figure 4:**
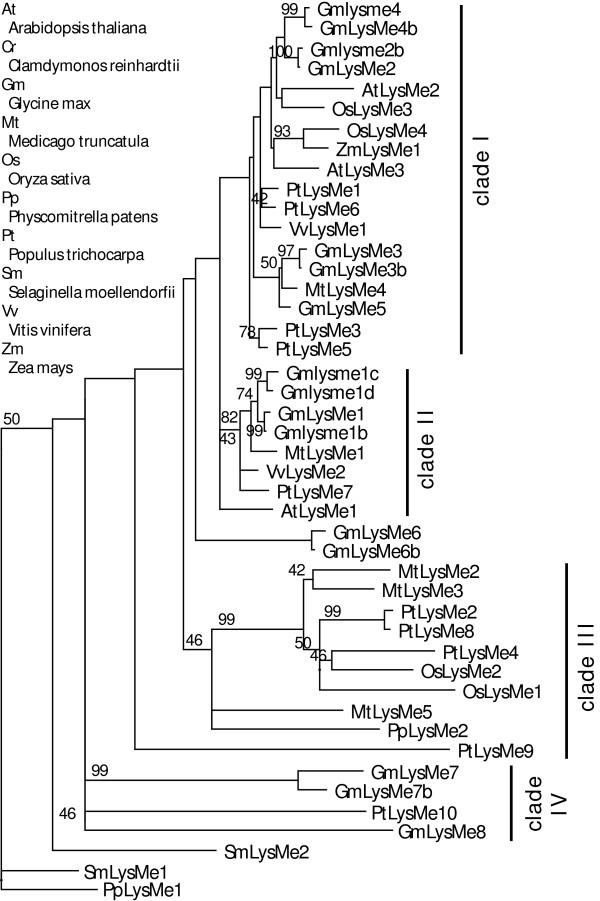
**Phylogenetic topology of plant LysMe proteins**. A majority-ruled parsimony tree with maximum-likelihood branch lengths was calculated using full-length LysMe sequences. Since LysMe proteins are generally small, the cutoff of the bootstrap values of 1000 independent trees was set to 40. The tree was rooted using a *Physcomitrella *sequence and a *Selaginella *sequence (see Additional file 3).

The LysMe proteins are about 100–120 aa in length and are the smallest group of LysM proteins in plants. All *LysMe *genes possess a very simple, intron-less gene structure. The LysMe proteins contain only one LysM motif without exception and the LysM motifs in LysMe proteins are the most conserved at the primary sequence level with an identity of 91% in a survey of 30 LysMe proteins from various plant species [2]. A total of 45 LysMe proteins from 9 species were grouped into 4 clades (Figure 4).

**Figure 5:**
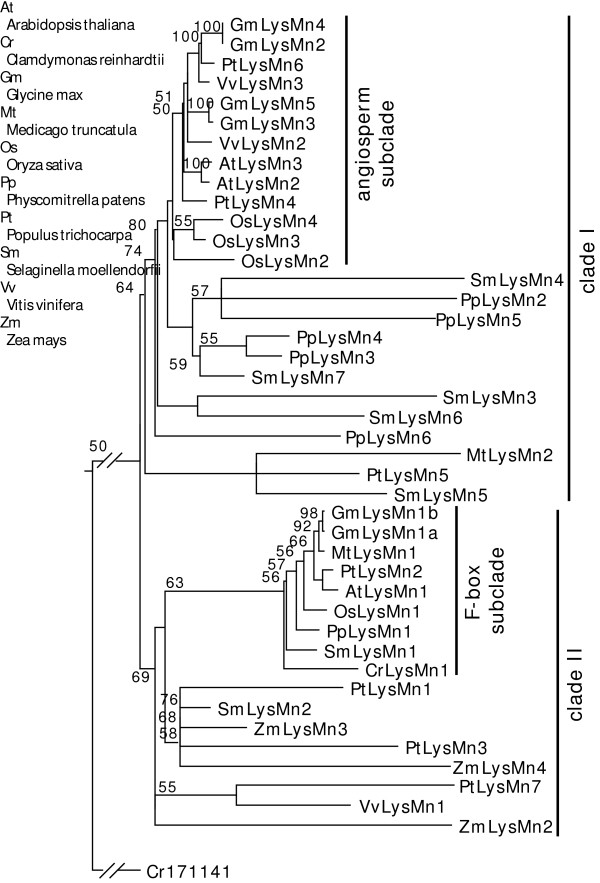
**Phylogenetic topology of plant LysMn proteins**. A majority-ruled parsimony tree with maximum-likelihood branch lengths was calculated using full-length LysMn sequences. Bootstrap values of 1000 independent trees larger than 50 are labeled on each branch. The tree was rooted using a green algal sequence, Cr171141.

A total of 41 LysMn proteins (including F-box+LysM proteins) from 9 species were grouped into 2 clades (Figure 5). Both clades can be divided into several subclades. Two distinctive subclades, angiosperm subclade and F-box subclade, were identified in each clade, respectively. The first subclade within the clade I contains sequences only from angiosperm plants and the average maximum-likelihood branch length is shorter than that of the other subclade, which contains mostly sequences from mosses. The first subclade within the clade II is a complete clade rooted with a green algae F-box+LysM (LysMn1) protein and contains LysMn1 proteins from all major lineages of land plants. Together with the fact that LysMn1 is a single-copy gene in most genomes (Figure 1), this implicates a conservation of their biological function. Similarly the F-box subclade has a shorter maximum-likelihood branch length than the other subclade.

### Microsynteny of orthologous LysM genes

**Figure 6:**
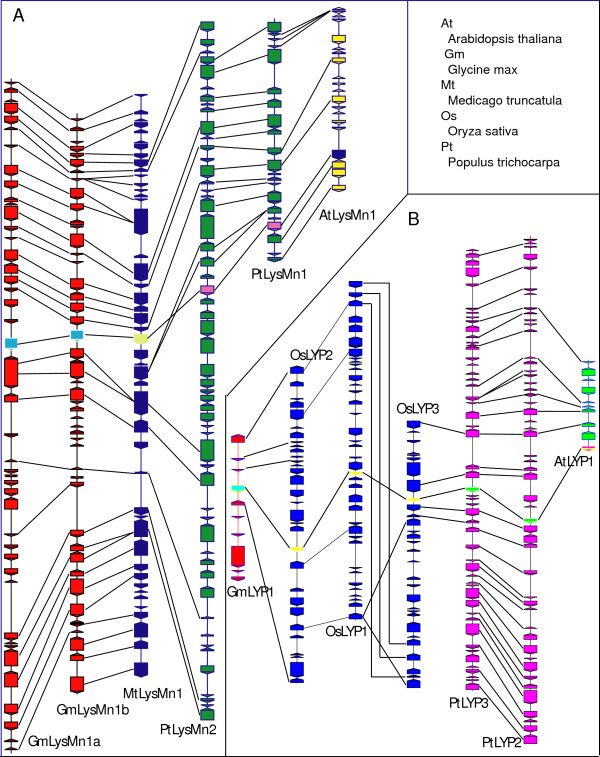
**Collinearity of genomic blocks surrounding plant *LysMn1 *(A) and *LYP *(B) genes**. Genes are shown in boxes with orientations indicated by a triangle. The blocks are colored differentially for each plant species. Plant *LysMn1 *and *LYP *genes are shown in inverted colors in each block. Orthologous genes are connected using solid black lines.

Phylogenetic topology is useful in deciphering gene orthology or paralogy, but is not sufficient in most cases. Collinearity of genes (or "microsynteny") across different species can be used as supporting evidence. Indeed, a total of 11 orthologous blocks of *NFR5*-like genes from 6 angiosperm species showed extensive microsynteny. A degraded microsynteny was also identified in *NFR1*-like orthologous regions in legume species [2]. This suggests that the functionally related *NFR5 *and *NFR1 *genes derived from a single duplicated ancestral genomic region. We identified additional regions of microsynteny for the *LYP*, *LysMe *and *LysMn *genes. Extensive microsynteny exists for LysMn1 (F-box+LysM) orthologous regions in the soybean, *Medicago*, poplar, and Arabidopsis genomes (Figure 6a), suggesting a genome conservation that mirrors the high sequence conservation of these proteins (Figure 5). OsLYP1, also known as the rice chitin elicitation binding protein (OsCEBiP), is the only LYP to which a biological function has been assigned [8]. It is capable of directly binding of chitin and was implicated in chitin-triggered immune responses. Microsynteny was observed in OsLYP1 orthologous regions in the rice, soybean, poplar and Arabidopsis genomes (Figure 6b), suggesting a possible conservation of function across these species.

### *LYK *gene duplications and genome polyploidy

It has been estimated that most, if not all, flowering plants, have one or more genome duplication events in their history [17-19]. The great expansion of the *LYK *gene family (Figure 1 and 2) is likely due to two rounds of independent genome doubling events followed by functional speciation. The LYK phylogenetic topology clearly reveals several rounds of gene duplications, especially the duplication event giving rise to the clades I and II (since the subclades IA and IIA are tandemly arrayed in their genomes). However, little is known regarding the timing of these duplications. In order to better understand the plant genome doubling events, we inferred the recent chronological order of these *LYK *duplication events by measuring the synonymous nucleotide substitutions per site (Ks) of duplicated genes, which is a common practice in dating gene duplication events. [18-23].

**Figure 7:**
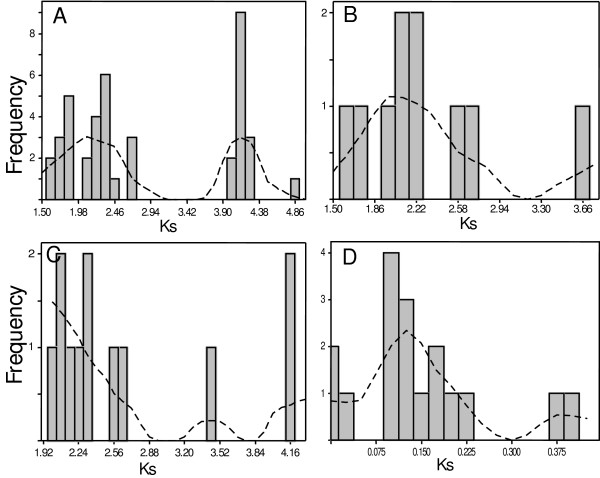
**Histogram plots of pairwise synonymous distance of *LYK *genes and *LysM *genes in soybean**. Y-axis denotes the frequency and x-axis denotes the synonymous distance (Ks). A: LYK clade I vs II; B: LYK subclade IA vs IB; C: subclade IIA vs IIB; D: homeologous *LysM *genes in soybean. The dash lines in each panel are the smoother lines. The peaks of the smoother lines indicate the relative timing of diverging events.

The clade-clade pairwise synonymous distance estimates involving clades III to V produce Ks values averaging greater than 4.0 (data not shown), indicating that they are likely the results of very ancient duplications and, therefore, very difficult to accurately predict. Therefore, we focused on the duplications between clades I and II and between the subclades VIA and VIB (Figure 2). Pairwise synonymous estimates between clades I and II revealed two peaks at 2.2 ± 0.2 and 4.2 ± 0.2, respectively (Figure 7a), suggesting two separate gene duplications, consistent with two genome duplications. The second peak at 4.2 ± 0.2 probably reflects the split between clades I and II and is unusually high. The duplication peaked at 2.2 ± 0.2 probably reflects the split between paralogous subclades (i.e., IA-IB and IIA-IIB) and is shared by flowering plants. Indeed, pairwise estimates between paralogous subclades IA-IB and between IIA-IIB both have a peak centered at 2.2 (Figure 7b, c). These suggest that the splits between IA-IB and IIA-IIB, observed in both monocot and dicot plants (Figure 2), likely resulted from the same genome duplication event. This split is assumed to have occurred at approximately 300 million years ago (mya) assuming a constant rate of 6.1 synonymous substitution per site every one billion years [20]. Consistent with this, ancient duplications in Arabidopsis were reported within this same time window [21,22]. The subclades IA and IIA are tandemly arrayed together in a head-to-head manner (Figure 2). This arrangement could have resulted from either a tandem gene duplication or from a whole genome duplication followed by local gene rearrangements. However, the tandem array of the subclades IA and IIA was observed only in Rosid plants. The split between the subclades IA and IIA, estimated at approximately 300 mya, appears to have occurred earlier than the emergence of dicot plants, which was estimated at 170–235 mya [24]. This suggests that the common ancestors of the subclades IA and IIA were not tandemly duplicated but were tandemly arrayed together likely through a large-scale genome rearrangement shortly before the emergence of Rosid plants. The subclades VIA and VIB are also tandemly arrayed in legume and *Vitis *genomes (Figure 3). The median value of pairwise estimates between VIA-VIB is 0.73 (data not shown) and is close to the values of 0.71 reported by Schlueter et al. (2004) and of 0.57 reported by Pfeil et al. (2005). This peak likely reflects a recent legume genome doubling event estimated at 54 mya [19,23,25].

We noticed 16 pairs of soybean *LysM *genes from the four phylogenetic topologies (Figure 2, 3, 4 and 5) that are highly similar at the protein sequence level (see Additional file 1). These likely represent homeologous gene pairs resulting from the most recent genome doubling event in soybean. The pairwise synonymous distances between these homeologous pairs fall within 0–0.40 and have a peak at 0.13 ± 0.03 (Figure 7d). This is close to the value of 0.188 reported by Schlueter et al. (2005). Assuming a rate of 6.1 synonymous substitutions per site every one billion years [20], the values for the LysM genes estimate this duplication event at approximately 14 mya, which is consistent with previous studies [19,23,25].

## Discussion

### LysM protein domain architectures

The LysM motif is a ubiquitous protein module found in association with a wide variety of protein domains, thus creating a tremendous diversity of domain architectures. Interestingly, only a simple core LysM architecture is conserved across different kingdoms and each individual kingdom has its own characteristic architectures (Figure 1), suggesting that LysM genes in different kingdoms have undergone distinct evolution and diversification, presumably reflecting functional selection. Recently, Onaga and Taira (2008) reported the isolation of one LysM protein (PrChiA) from the fern plant, Pteris ryukyuensis, consisting of 2 LysM motifs and a glycol_hydro_18 domain [26]. However, our analysis finds this specific protein architecture is limited to the fungal kingdom. The 2 LysM motifs of this protein are 95% identical to each other, in contrast to the fact that 2 LysM motifs in a given plant LysM protein are usually different from each other. Moreover, majority-ruled parsimony topology based on LysM motif sequences (about 42 aa in length) clearly shows that the 2 LysM motif sequences of PrChiA are separated from plant LysM motif sequences and lie in the same clade with fungal LysM motif sequences (see Additional file 2). It is not uncommon to isolate fungal genes from RNA preparations derived from plants and, therefore, further study of the origin of this proposed fern gene is warranted. This case exemplifies the value of domain architecture and phylogenetic studies as an aid to predict gene function and distribution.

Although LysM proteins presumably bind oligomers of N-acetyl glucosamine (e.g., chitin) and peptidoglycan, it is not surprising to find the LysM motif associated with other carbohydrate-binding modules given the fact that a complex array of carbohydrates exists in nature. These carbohydrate-binding modules include the B_lectin domain , chitin-binding domain in chitinases, and WSC (cell-wall integrity and stress response component) domain [27]). The presence of these domains with the LysM domain is consistent with the pattern that these proteins are involved in binding polysaccharides, including those of complex structure.

*Physcomitrella*, a bryophyte and arguably the most primitive plant whose genome has been sequenced, possesses a few plant-specific LysM architectures. The *LYK *genes, one example, are consistent with the recent report of a number of receptor-like kinases in liverworts [28]. Proteins with a LysM domain and predicted F-box domain are highly conserved in plant kingdom and may play a role in regulating the stability of yet unknown protein substrates. It is interesting to speculate that these are glycoproteins recognized by the LysM domain. Interestingly, this domain arrangement is also present in the *Chlamydomonas *genome, the green algae that diverged from land plants over 1 billion years ago. This indicates that the role for these proteins, presumably in glycoprotein degradation, can be traced back in *Chlamydomonas *and other primitive species as well. This notion is supported by the fact that green algae and moss genomes contain ubiquitin-proteasome elements, such as ubiquitin, ubiquitin-conjugating genes, and other F-box genes (; ).

A few architectures that are present exclusively in primitive plants were progressively and apparently permanently lost during angiosperm evolution (the lack of fern and gymnosperm genome sequences precludes us from examining this trend in these lineages). Among these lost architectures, two of them, peptidase+LysM and C2+LysM, are also present in the genome of *Chlamydomonas*, suggesting the existence of common ancestors for these LysM proteins in nature. These LysM proteins appeared to have been lost during the change from the unicellular, aquatic life style to a terrestrial, vascular life style. However, the nature of the peptidase domain in *Chlamydomonas *(peptidase_C1) is different from that in *Physcomitrella *(peptidase_M23). Actually, LysM+peptidase_M23 proteins are present in cyanobacteria (data not shown) and, therefore may have been introduced into primitive plants by horizontal gene transfer.

### *LysM *gene evolution and plant genome duplications

Only one copy of F-box+LysM genes was retained in most plant genomes after many rounds of polyploidy events. Similarly, the copy numbers of *LysMn *and *LYP *genes remain roughly the same across different plant genomes. These suggest that increased gene copy number may have deleterious effects and the dosage of these genes is under tight regulation. In contrast, the *LYK *gene family underwent several successive rounds of expansion, especially in flowering plants. Indeed, the expansion of the *LYK *gene family can be found in primitive plant lineages; that is, from the 2 *LYK *genes in *Physcomitrella *(bryophytes) to 5 *LYK *genes in the *Selaginella *(lycophytes) genome (Figure 1). This pattern of differential expansion of *LysM *genes is probably due to the different rates of gene duplications of individual *LysM *gene families in plants. However, it is more likely that this is due to the different rates of gene retention following gene duplications. For example, *LYK *genes, compared to other *LysM *gene families, appear to have been retained more frequently following gene duplications.

The phylogenies of *LysM *genes, especially *LYK *genes, reveal several rounds of gene duplication, consistent with whole genome duplications (Figure 2, 3, 4 and 5). The timing of these duplications can be estimated by the accumulation of synonymous to non-synonymous changes in the sequences. Pairwise synonymous distance shows that the LYK subclades VIA and VIB likely resulted from the large-scale gene duplication shared by legume plants estimated at 54 mya [19,23,25]. Genome duplications around the time window of 50 mya were also reported in other flowering plants including grasses and Solanaceae plants [23]. Pairwise synonymous estimates suggest that the splits between the LYK subclades IA and IB and between IIA and IIB may also be the outcome of the same round of large-scale duplication estimated at 300 mya (Figure 7). The Arabidopsis genome appears to have undergone ancient genome duplications around this time window [21,22]. Moreover, our data suggest that this genome wide duplication is shared in flowering plants and may have pre-dated the divergence of gymnosperm and angiosperm plants ~300 mya [29].

A few LYK subclades are tandemly arrayed in plant genomes (Figure 2). These arrangements could arise from either a local tandem duplication or a genome doubling followed by local rearrangement. The split of subclades IA and IIA was estimated at 300 mya, probably upon or shortly after the divergence of gymnosperm and angiosperm plants [29]. However, the tandem array pattern is observed only in Rosid plants (Figure 2). This pattern may be conserved in dicot plants but, lacking the data from Asterids and Caryophyllids, a firm conclusion is not possible. The fact that the gene duplication likely occurred earlier than the emergence of eudicot plants favors the hypothesis that the common ancestors of the subclades IA and IIA resulted from a genome duplication with their current tandemly arrayed positions arising later due to local rearrangement, The data do not support a conclusion that the common ancestors of subclades IA and IIA resulted from local tandem duplication and were de-associated in monocots and re-associated again in dicots. In contrast, the data do suggest that the tandemly arrayed VIA and VIB resulted from a recent large-scale duplication estimated at 54 mya.

### Functional characterization of *LysM *genes

The LysM domain was first described in bacterial enzymes whose role is to degrade the peptidoglycan cell wall [6,7]. In this case, the LysM domain anchors these enzymes to the peptidoglycan, a polymer structurally similar to chitin. Hence, the discovery of the LysM domain in proteins genetically identified as the receptor for the bacterial chitooligosaccharide nodulation factor immediately suggested that the LysM domain mediated interaction with the oligosaccharide [9-13]. Such a role is also consistent with the finding of the LysM domain in LYKs or LYPs implicated in the recognition of chitin, a well known fungal PAMP, which elicits the plant innate immunity response [8]; [9,10]. These data clearly suggest a similar oligosaccharide binding role for the LysM domains found in other LysM protein families. Recently, peptidoglycan was shown to trigger typical PAMP-elicited immune responses in plants [2], while chitin was identified as a PAMP active on animal cells [30,31]. It is very likely that LysM domain containing receptors are involved in mediating these responses.

### Uniformity of *LysM *gene nomenclature

A total of 201 *LysM *genes were identified from 10 plant species in this study. Considering that *LysM *genes are ubiquitous in the plant kingdom, one could imagine that innumerable LysM genes exist in plants. Naming these genes could be a great challenge. Indeed, there are already a diversity of names given to various *LysM *genes [8-11,13,32] and this creates confusion within the research community. In order to reduce this confusion, we incorporated all reported names of *LysM *genes in the phylogeny (Figure 2 and 3). Previously, we proposed a uniform nomenclature for all LysM domain containing proteins [2] and we used this same nomenclature in this study. This nomenclature system reflects subcellular localizations, phylogenetic relationships, and biological functions. We recommend adoption of this nomenclature as a way to reduce confusion in anticipation of an increasing interest in this important group of plant proteins.

## Conclusion

We report a domain architecture catalogue of LysM proteins across all kingdoms and describe a comparative and evolutionary genomics study of *LysM *genes in the plant kingdom. Our data show that LysM-containing proteins display a wide range of domain architectures. Each individual kingdom appears to have evolved a distinct array of domain architectures, suggesting the presence of distinctive evolutionary paths in individual kingdoms. We show that early plant lineages acquired four characteristic domain architectures and progressively lost several primitive domain architectures. Apparently, *LYK *gene family underwent intensive expansion, while the dosages of other types of plant *LysM *genes roughly stay the same throughout the entire plant kingdom. One of the two groups of tandemly arrayed plant *LYK *genes likely resulted from an ancient genome duplication followed by local genomic rearrangement, while the origin of the other groups of tandemly arrayed *LYK *genes remains obscure. We defined the orthologous and paralogous relationships of plant *LysM *genes based on sequence alignment, phylogenetic topology, microsynteny, and nucleotide substitution levels. We also identified 16 pairs of putative homeologous genes in soybean. This study will provide clues to functional characterization of plant *LysM *genes and be informative to functional studies of animal *LysM *genes.

## Methods

### Sequence mining

The mining of LysM sequences was performed as previously described [2]. The genome databases in this study are: Arabidopsis (*Arabidopsis thaliana*; ); rice (*Oryza sativa*; ); maize (*Zea mays*; ); poplar (*Populus trichocarpa*; ); *Medicago truncatula *; *Lotus japonicus *; *Vitis vinifera *; soybean (*Glycine max*; ); pine (*Pinus taeda*; ); moss (*Physcomitrella patens*; ); spikemoss (*Selaginella moellendorffii*; ); green algae (*Chlamydomonas reinhardtii*; ). The final LysM proteins sequences, CDS sequences, LysM positions, gene and intron structures, and other predicted features were compiled into the Additional file 3.

### LysM domain architectures

The LysM domain architectures were extracted from the Pfam database . For architectures in the prokaryotic, fungal and animal kingdoms, only those identified in more than 5 sequences were kept to draw the diagram of LysM domain architectures (Figure 1). The domain structures of all plant LysM proteins were analyzed with Pfam  and inter-ProScan . Signal peptides and transmembrane domains were predicted using SignalP  with both Neutral Network and Hidden Markov Models and TMHMM , respectively.

### Alignment, phylogeny, and synonymous distance

Protein sequences were aligned using MUSCLE3.6 [33] with a fasta output format and manually edited using Jalview [34]. Majority-ruled parsimonious trees were generated using the program protpars of PHYLIP [35] with maximum likelihood branch lengths calculated using TREE-PUZZLE [36]. Bootstrap values were calculated using the program seqboot of the PHYLIP package. All trees were viewed and printed into a pdf format using A Tree Viewer [37]. For calculation of synonymous distance, codon-aligned nucleic acid sequences were created using RevTrans 1.4 . Synonymous nucleotide substitutions per site were calculated using the program yn00 of the PAML package [38]. The histograms of calculated Ks values were plotted and descriptive statistics were displayed using the Minitab 15.0.

### Microsynteny analyses

Genomic sequences surrounding selected LYP and LysMn genes, about 1–2 Mb in length, were extracted from the above databases. These stretches of genomic sequences were annotated using a dicot species model and Arabidopsis matrix of FGENESH for dicot plants and a monocot species model and rice matrix for rice. The annotated protein sequences were compiled together into a peptide sequence database using the BLAST program. Repetitive sequences were excluded from the databases. BLASTp was used to compare protein identity and similarity against the database with an *E*-value cutoff of 1e-20 and a percent identify cutoff of 35% between species and 40% within the same species. The gene and intron symbols were drawn using GenePicPipe Synteny Grapher . The microsynteny maps were finally drawn in Adobe Illustrator 10.0.

## Authors' contributions

XZ, SBC, and GS conceived this study. XZ collected data, carried out analyses and wrote the draft manuscript. SBC contributed to data analyses. SBC and GS discussed the results and contributed to manuscript revisions. All authors read and approved the final manuscript.

## Supplementary Material

Additional file 1 **The sequence comparisons of homeologous LysM genes in soybean**. This Table shows the identity and similarity of pairwise comparisons of homeologous *LysM *genes in soybean. Click here for file

Additional file 2 **The LysM motifs of Pteris ryukyuensis chitinase A (PrChiA) are associated with fungal LysM motifs**. A majority-ruled parsimony tree with maximum-likelihood branch lengths was calculated using selected plant LysM motif sequences and fungal LysM motifs in glycol_hydro_18 proteins. Bootstrap values of 1000 independent trees larger than 40 were labeled on each branch. The fungal sequences were extracted from the Pfam database. Click here for file

Additional file 3 **The peptide and CDS sequences of *LysM *genes in plants**. This excel file contains not only the peptide and CDs sequences of plant LysM genes but also the collection of common features of plant *LysM *gene in many aspects. Click here for file
