# Supplementary material for: Evolutionary genomics of LysM genes in land plants
Source: BMC Evol Biol. 2009 Aug 3;9:183. doi: 10.1186/1471-2148-9-183 (PMC2728734; doi:10.1186/1471-2148-9-183)
Supplement: Additional file 1 — The sequence comparisons of homeologous LysM genes in soybean. This Table shows the identity and similarity of pairwise comparisons of homeologous LysM genes in soybean. [file 1471-2148-9-183-S1.doc]

| S. Table 2. Sequence comparisons of homeologous *LysM* genes in soybean. | |
| --- | --- |
| GmLysM | Identity/similarity |
| GmNFR1a-GmNFR1b | 99/99 |
| GmNFR5a-GmNFR5b | 94/96 |
| GmLYK4-GmLYK7 | 93/95 |
| GmLYK6-GmLYK6b | 88/92 |
| GmLYK9-GmLYK9b | 95/96 |
| GmLYK10-GmLYK10b | 89/93 |
| GmLYK11b-GmLYK11c | 97/98 |
|  |  |
| GmLysMe1- GmLysMe1b | 90/93 |
| GmLysMe1c- GmLysMe1d | 82/86 |
| GmLysMe2- GmLysMe2b | 94/95 |
| GmLysMe3- GmLysMe3b | 93/96 |
| GmLysMe4- GmLysMe4b | 93/95 |
| GmLysMe6- GmLysMe6b | 91/96 |
|  |  |
| GmLysMn1a-GmLysMn1b | 91/95 |
| GmLysMn2-GmLysMn4 | 99/99 |
| GmLysMn3-GmLysMn5 | 84/86 |
