# Supplementary material for: Evolutionary genomics of LysM genes in land plants
Source: BMC Evol Biol. 2009 Aug 3;9:183. doi: 10.1186/1471-2148-9-183 (PMC2728734; doi:10.1186/1471-2148-9-183)
Supplement: Additional file 2 — The LysM motifs of Pteris ryukyuensis chitinase A (PrChiA) are associated with fungal LysM motifs. A majority-ruled parsimony tree with maximum-likelihood branch lengths was calculated using selected plant LysM motif sequences and fungal LysM motifs in glycol_hydro_18 proteins. Bootstrap values of 1000 independent trees larger than 40 were labeled on each branch. The fungal sequences were extracted from the Pfam database. [file 1471-2148-9-183-S2.pdf]

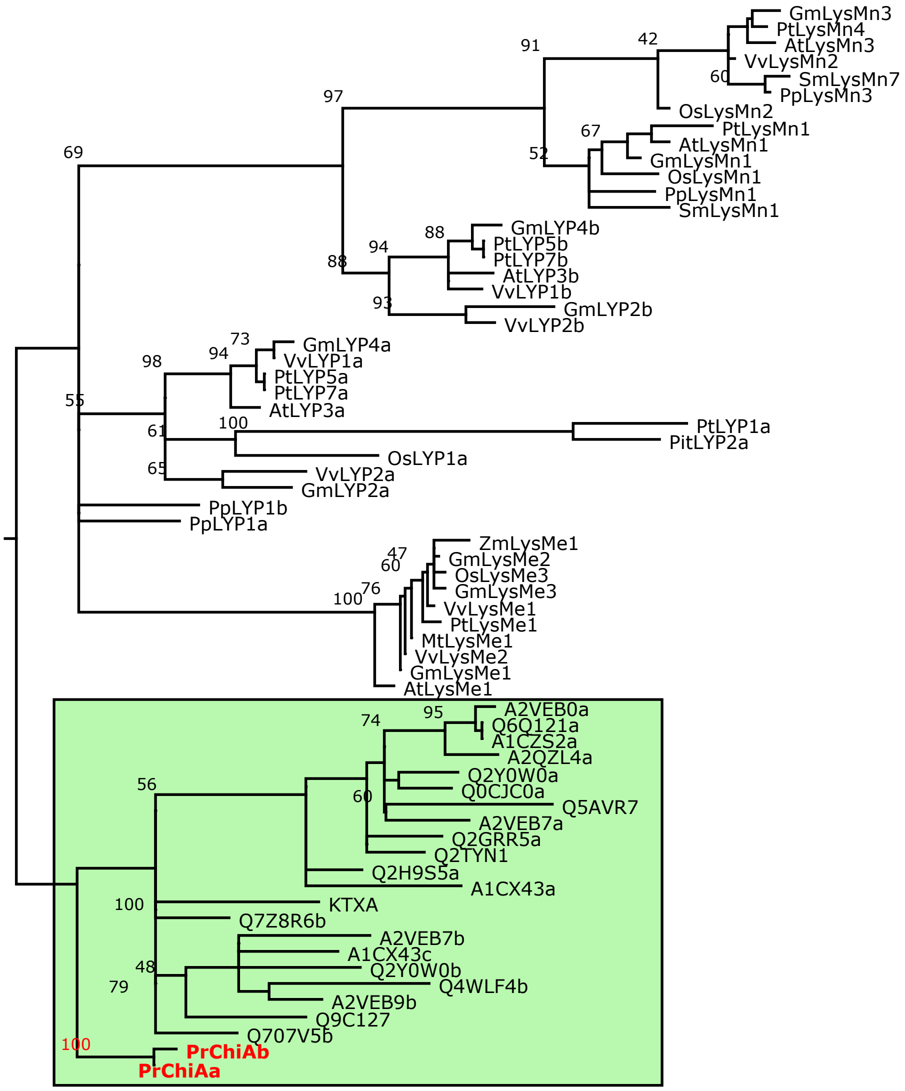

Figure S1
Zhang et al.

Figure S1. The LysM motifs of *Pteris ryukyuensis* chitinase A (PrChiA) are associated with fungal LysM motifs. A majority-ruled parsimony tree with maximum-likelihood branch lengths was calculated using selected plant LysM motif sequences and fungal LysM motifs in glycol_hydro_18 proteins. Bootstrap values of 1000 independent trees larger than 40 were labeled on each branch. The fungal sequences were extracted from the Pfam database.
